# Propensity score modeling of adolescent e-cigarette use and cognitive performance: One-year follow-up study

**DOI:** 10.18332/tid/216705

**Published:** 2026-03-05

**Authors:** Hongying Daisy Dai, Troy B. Puga, Jiani Zhang, Neal L. Benowitz

**Affiliations:** 1 College of Public Health, University of Nebraska Medical Center, Omaha, United States; 2 Department of Orthopedic Surgery, HCA Medical City Healthcare UNT-TCU GME, Denton, United States; 3 Division of Cardiology, Department of Medicine, University of California, San Francisco, United States

**Keywords:** propensity score, e-cigarette use, neurocognition, the adolescent brain and cognitive development (ABCD) study, NIH toolbox

## Abstract

**INTRODUCTION:**

Adolescent e-cigarette use remains an important public health challenge, and evidence on its neurocognitive effects at an early age is limited. This study examined associations between exclusive e-cigarette use and cognitive performance in adolescents.

**METHODS:**

This study is a propensity score modeling cohort study. This study performed a secondary data analysis on data collected from 21 US sites in the Adolescent Brain Cognitive Development (ABCD) Study between 1 October 2016 and 31 October 2018, with one year of follow-up data. Adolescents reported substance use at ages 12–13 years and completed National Institute of Health (NIH) Toolbox Cognition Batteries one year later, which consists of the following scores: Picture Vocabulary, Flanker Inhibitory Control and Attention, List Sorting Working Memory, Dimensional Change Card Sort, Pattern Comparison Processing Speed, Picture Sequence Memory, and Oral Reading Recognition. Propensity score models incorporating 12 confounders assessed associations between baseline exclusive e-cigarette use (vs no tobacco use) and follow-up cognitive performance at one year.

**RESULTS:**

The cohort included 4574 participants (55.7% White, 25.3% Hispanic, 8.9% Black). Propensity score matching substantially improved covariate balance between e-cigarette users and non-users. Adolescents who exclusively used e-cigarettes in the past six months at baseline (n=35) had lower scores in Oral Reading Recognition (b[Standard Error (SE)]= -4.5 [1.3]; 95% CI: -7.3 – -1.7; p=0.003) and similar associations were observed in multivariable regression models adjusting for demographic, socioeconomic, and behavioral covariates.

**CONCLUSIONS:**

Exclusive e-cigarette use among early adolescents was associated with poorer performance in specific cognitive domains. These preliminary findings raise concerns about potential neurocognitive implications of e-cigarette use and warrant confirmation in larger, longitudinal studies with longer follow-up.

## INTRODUCTION

Adolescence represents a critical stage with rapid improvement in cognitive performance and brain maturation^1^. Youth e-cigarette use persists as a modern public health challenge. Since 2014, e-cigarettes have been the most commonly used tobacco product among youth in the US, with 10.0% of high school (1.56 million) and 4.6% of middle school (550000) students reporting current (past 30-day) use in 2023^2^. In contrast, cigarette smoking among adolescents has markedly declined, with 1.6% of high school students reporting cigarette smoking in the past 30 days in 2023, according to the National Youth Tobacco Survey^2^. E-cigarette products have targeted children using social media and providing a wide variety of flavors appealing to youth. Some children are also susceptible to e-cigarette initiation due to low media literacy about e-cigarette marketing and unawareness of the potential side effects of e-cigarettes^3^.

E-cigarettes might pose adverse health effects on the users, with the full effects over time still relatively unknown. E-cigarette aerosol contains a number of potentially toxic substances (e.g. carbonyl compounds, heavy metals, and flavorings) and vaping is associated with the development of nicotine dependence and an increased risk of respiratory symptoms^4^. Furthermore, the current generations of vaping products often contain high concentrations of nicotine in a salt formulation – which permits the delivery of substantial nicotine amounts through a palatable aerosol^5^. Acute nicotine exposure provides brain reward through its effects on dopamine release. Chronic nicotine exposure increases the number of central nervous system nicotine acetylcholine receptors (nAChRs) in animals and human smokers *in vivo*^6^.

Since the use of e-cigarettes remains a relatively recent behavior, longitudinal studies that examine the neurocognitive effects of e-cigarette use in the adolescent population are lacking. Previous literature has shown that tobacco (including cigarettes, e-cigarettes, cigars, etc.) initiation and use in ever tobacco users at the age of 9–10 years is associated with lower neurocognition through the use of neuroimaging measures and the NIH Toolbox Cognition Battery^7^. Ever use might represent experimental tobacco use behaviors, and studies need to expand our knowledge on the effects of exclusive e-cigarette use over the recent period (e.g. the past 6 months). The NIH Toolbox Cognition Battery is a set of brief, psychometrically validated measures to assess language, memory, and executive function^8^. The tasks are selected based on a consensus-building process and developed and validated using item response theory and computerized adaptive testing with high reliability^9^. These skills are critical in childhood education and learning and are another reason longitudinal assessment is necessary.

Based on the findings from animal studies relating nicotine and brain development, we sought to investigate whether e-cigarette use is associated with cognitive performance outcomes^6,7^. The present study analyzed the data from the Adolescent Brain and Cognitive Development (ABCD) Study^10^ to assess associations between exclusive e-cigarette use and subsequent neurocognitive performances, measured by the NIH Toolbox Cognitive Battery one year later.

## METHODS

### Data and participants

This study performed secondary data analysis on the ABCD 5.0 Data released by the National Data Archive (NDA). The ABCD Study is a large cohort that enrolled children aged 9–10 years across 21 US research sites between 1 October 2016 and 31 October 2018^11^. Youths were recruited through a probability sample of schools selected for sex at birth, race/ethnicity, socioeconomic status, and urbanicity. Participants are assessed by a comprehensive battery including clinical interviews, surveys, neurocognitive tests, and neuroimaging longitudinally. All parents or guardians provided written informed consent, and children gave written assent^12^.

This study utilized the substance use data measured at the ABCD Study Year 3 (2019–2021) as exposure variables when adolescents were aged 12–13 years and NIH Toolbox cognition battery data measured at Year 4 (2020–2022) as outcome variables when adolescents were aged 13–14 years. This study focused on Years 3 and 4 of the ABCD cohort, as earlier waves showed very low prevalence of e-cigarette use, limiting their utility for examining associations with cognitive outcomes^13^. The ABCD study procedure was approved by the centralized institutional review board (IRB) of the University of California, San Diego, and by the IRB at each local institution. This report was approved by the University of Nebraska Medical Center Institutional Review Board (0468-21-EX) and follows the Strengthening the Reporting of Observational Studies in Epidemiology (STROBE) guidelines for cohort studies^14^.

### Measures

Past 6-month e-cigarette use at the age of 12–13 years was the exposure variable at baseline. A detailed, web-based Timeline Follow-Back (TLFB) interview^15^ was administered to assess e-cigarette and other tobacco use since the participant’s last visit (approximately one year). We created a variable to measure the past 6-month e-cigarette use (yes, no) if participants reported use of e-cigarettes on ≥1 day in the past 6 months. Similarly, we created the past 6-month use of cigarettes, smokeless tobacco, cigars, hookah, and pipe tobacco. Those who reported past 6-month use of e-cigarettes but not use of other tobacco products were classified as exclusive e-cigarette users in the past 6 months, and those who reported no use of any tobacco products were classified as non-tobacco users in the past 6 months (non-exposed group). We focused on exclusive e-cigarette use to minimize confounding from other tobacco products, as dual use could obscure the specific impact of e-cigarette exposure on cognitive function^16^. The sensitivity analysis includes the number of e-cigarette use days, ranging from 1 to 180, as assessed by the TLFB among youth users, with a value of 0 assigned to non-users.

NIH Toolbox Cognition Battery (outcome variables at follow-up after 1 year) was administered through an iPad, and tasks were completed within 35 minutes (see 6 tests in Supplementary file Appendix 1). Patients with missing NIH Toolbox Cognition Battery data at follow-up were excluded from analysis.

Covariates measured at age of 12–13 years included: 1) age; 2) self-rated puberty development scale (PDS)^17^, which consists of five questions regarding changes in height, body hair, skin, voice, and facial hair (males) or breast development and menarche (females). The PDS is a four-point Likert scale where a higher average score (ranging from 1=had not begun to 4=already complete) indicates a more advanced level of perceived physical development; 3) child-reported parent monitoring scale (5 items, e.g. ‘How often do your parents know where you are?’, Cronbach’s alpha=0.50)^18^. Higher scores indicate stronger parental monitoring; and 4) Past 6-month marijuana use. Due to the popularity of vaping marijuana among adolescents, we also included a variable to measure marijuana use based on the TLFB, which assessed different marijuana use modes, including smoked marijuana flowers, blunts, vaped marijuana flowers, edible marijuana, smoked marijuana concentrates, marijuana-infused drinks, marijuana tincture, and synthetic marijuana. Those who reported use of ≥1 marijuana mode in the past 6 months were classified as past 6-month marijuana users.

Covariates measured at the beginning of the ABCD study included sex at birth (males, females), race/ethnicity (non-Hispanic White, non-Hispanic Black, Hispanic, Other), parental education level (i.e. high school graduates or lower, some college, college graduates or higher) and parental income ($) level (<50000; 50000–99999; or ≥100000). Ever tobacco use was measured by children self-reported ever use of tobacco products, including e-cigarettes, cigarettes, cigars, smokeless tobacco, hookah, pipe, and nicotine replacement^15^. Vaping frequency (the number of days e-cigarettes used in the past 6 months) as well as history of substance use, school environment factors, and child mental health assessed according to DSM-5 criteria were also used as covariates in sensitivity analysis.

### Statistical analysis

Weighted participant characteristics were reported, overall and by e-cigarette use status. Rao-Scott χ^2^ test and linear regression models were conducted to detect group differences for categorical and continuous variables, respectively. To investigate the longitudinal associations between baseline exclusive e-cigarette use and subsequent cognitive performance, we conducted propensity score modeling (PSM) to adjust for 12 confounding variables in the primary analysis. Following the reporting and analytical guidance^19^, the details of PSM can be found in Supplementary file Appendix 3. In the secondary data analysis, separate multivariable linear mixed models were performed to examine the associations of e-cigarette use at age of 12–13 years and NIH Toolbox Cognition Battery measured 1 year later, adjusted by a number of covariates, including age, sex at birth, race/ethnicity, youth pubertal stage, ever use of tobacco, past 6-month marijuana use, parental education level, parental income, and parent monitoring. Due to the complexity of confounding in the association between e-cigarette use and cognitive development, a broad range of covariates was selected *a priori* as potential confounders to account for sociodemographic, behavioral, environmental, and psychological influences (Supplementary file Appendix 2). In the PSM adjustment, we excluded vaping frequency because the non-exposed group had a value of zero, making it impossible to achieve balance between groups. Instead, we conducted a sensitivity analysis including vaping frequency (the number of days e-cigarettes used in the past 6 months) as well as history of substance use, school environment factors, and child mental health assessed according to DSM-5 criteria. These covariates were selected based on a review of the literature, established cognitive assessment protocols, and factors known to influence child cognitive development, brain growth, and patterns of tobacco use^7,20,21^. Both unadjusted and adjusted regression coefficient B and standard error (SE) were estimated using the Taylor series method in the variance-covariance matrix.

All analyses followed the statistical guide for the population-based analysis of the ABCD study to account for the clustering of participants over 21 study sites, sample selection biases, and non-responsiveness in the observational study design^22^. The weight variable was generated using a propensity model of age, sex, and race/ethnicity and missing data imputation to ensure that weighted ABCD data maintain the sample demographics in accordance with the American Community Survey 3rd- and 4th-grade enrollment statistics at each site^12^. Study sites were included as clusters in survey analytical procedures. Statistical analyses were conducted in SAS 9.4 (Cary, NC), and a p<0.05 was considered significant.

## RESULTS

**Figure 1:**
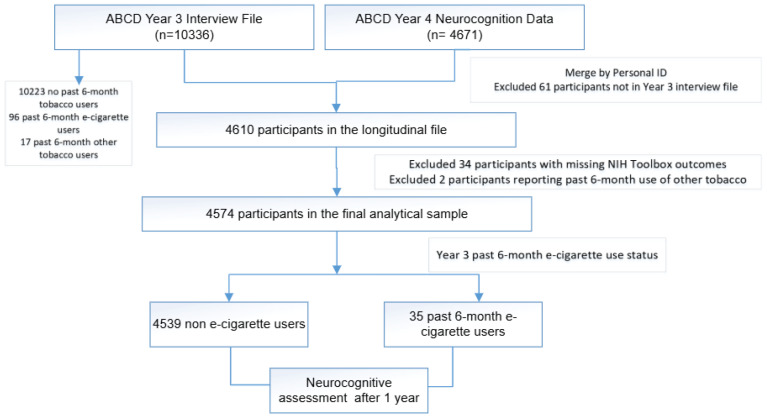
Flow chart of the analytical sample for a propensity score-weighted cohort study of exclusive e-cigarette use and neurocognitive performance, ABCD Study, Years 3–4, 2019–2022 (N=4574)

As illustrated in Figure 1, 10336 participants completed the substance use assessments, and 4671 completed the neurocognition tests in the following year. After excluding 61 individuals not present in the Year 3 interview file, the final combined longitudinal dataset comprised 4610 participants. We further excluded 34 subjects with missing NIH Toolbox outcomes and 2 subjects who reported use of other tobacco products in the past 6 months. The final analytical study sample consisted of 4574 participants, including 4539 non-tobacco users and 35 exclusive past 6-month e-cigarette users.

The overall sample (mean [SE] age: 12.9 [0.04] years) included 51.7% of males and 48.3% of females (Table 1); 55.7% of participants were White, 25.3% Hispanic, 8.9% Black, and 10.1% identified as other races. About 57.1% of participants had parents who were college graduates; 41.1% of participants had a parental income of <$50000. About 0.9% of participants had tobacco ever use, and 0.4% of participants reported use of marijuana in the past 6 months. Compared to non-tobacco users, past 6-month e-cigarette users tended to be older (mean difference=0.4 years; 95% CI: 0.1–0.7), have a lower parental monitoring scale (mean difference = -0.4; 95% CI: -0.7 – -0.2), and reported a higher prevalence of past 6-month marijuana use (risk difference=19.0%; 95% CI: 1.4–36.6). E-cigarette use status also differed by race/ethnicity and parental income level.

**Figure 2:**
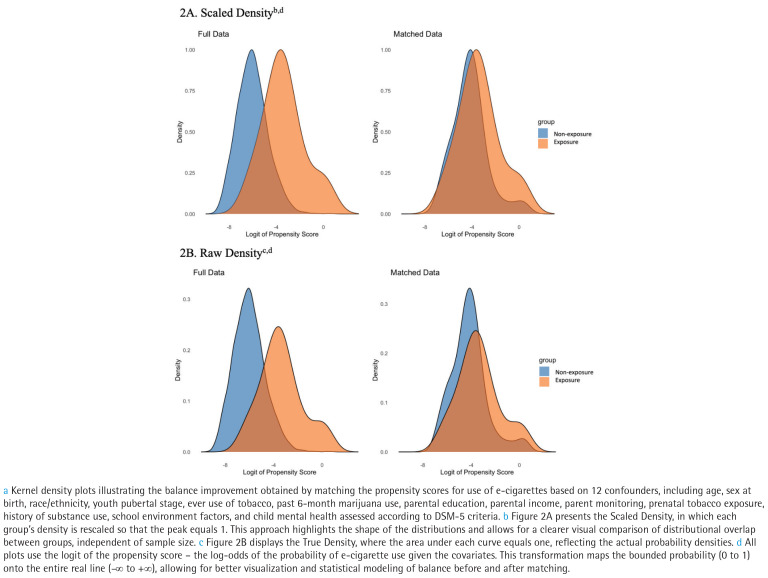
Density of logit of propensity score for full data and matched data of exclusive past 6-month e-cigarette use among adolescents, ABCD Study, Year 3, 2019–2021 (N=4574)^a^

As shown in Figure 2 and Supplementary file Appendix 4, propensity score weighting substantially improved balance between the two groups. In the original sample, the non-exposed group tended to have lower logit propensity scores than the test group. After weighting, the standardized mean difference for the logit propensity score dropped from 1.7 to 0.0, indicating a 99.9% reduction. Most covariates achieved acceptable balance, with standardized differences reduced by 66% to 100%. Key variables such as child age, substance use disorder symptoms (DSM-5), and parental monitoring showed large improvements.

**Table 1:** Weighted baseline characteristics of adolescents aged 12–13 years by past 6-month exclusive e-cigarette use status, ABCD Study, Year 3, 2019–2021 (N=4574)

*Characteristics*	*All ^a^*	*No use ^a^ (N=4539)*	*E-cigarette use ^a^ (N=35)*	*p ^b^*
**Age** (years), mean (SE)	12.9 (0.04)	12.9 (0.04)	13.3 (0.1)	0.01
**Puberty development scale,** mean (SE)	2.5 (0.03)	2.5 (0.03)	2.6 (0.1)	0.36
**Parent monitoring scale,** mean (SE)	4.4 (0.02)	4.4 (0.02)	4 (0.1)	0.001
**Sex**				0.71
Male	2398 (51.7)	2378 (51.7)	20 (54.7)	
Female	2175 (48.3)	2160 (48.3)	15 (45.3)	
**Race/ethnicity**				0.03
White	2590 (55.7)	2579 (55.9)	11 (35.2)	
Black	479 (8.9)	475 (8.9)	4 (6.7)	
Hispanics	942 (25.3)	930 (25.1)	12 (40.1)	
Other	560 (10.1)	552 (10.1)	8 (18.1)	
**Parental education level**				0.75
High school or lower	563 (14.8)	558 (14.8)	5 (19.9)	
Some college or associate degree	1102 (28.0)	1092 (28.0)	10 (29.6)	
College graduates	2909 (57.1)	2889 (57.2)	20 (50.5)	
**Parental income level** ($)				
<50000	1441 (41.1)	1427 (41.1)	14 (43.1)	0.002
50000–99999	1259 (29.9)	1243 (29.8)	16 (43.9)	
≥100000	1874 (29.0)	1869 (29.2)	5 (13.0)	
**Tobacco ever use**				
No	4537 (99.1)	4503 (99.1)	34 (96.6)	0.09
Yes	32 (0.9)	31 (0.9)	1 (3.4)	
**Past 6-month marijuana use**				<0.0001
No	4558 (99.6)	4529 (99.8)	29 (80.8)	
Yes	16 (0.4)	10 (0.2)	6 (19.2)	
**Prenatal tobacco exposure**				
No	3844 (86.2)	3815 (86.2)	29 (80.7)	0.33
Yes	608 (13.8)	604 (13.8)	4 (19.3)	

a Data are given as frequencies (n) with column weighted percentages (%) for categorical variables, and mean (standard error [SE]) for continuous variables. Sampling weights and site clustering were accounted for in all analyses.

b Rao-Scott χ^2^ test and linear regression models were conducted to detect group differences for categorical and continuous variables, respectively.

Table 2 presents the prospective associations between exclusive e-cigarette use and subsequent NIH Toolbox Cognition Battery. In the PSM analyses, baseline e-cigarette users exhibited lower Oral Reading Recognition (adjusted b [SE]= -4.5 [1.3]; 95% CI: -7.3 – -1.7; p=0.003) and Picture Vocabulary Test (adjusted b [SE]= -5.5 [0.7]; 95% CI: -7.1 – -3.9; p<0.0001) scores at follow-up after 1 year, than non-tobacco users. The Picture Sequence Memory score was lower among e-cigarette users (vs non-tobacco users) in the unadjusted model (unadjusted p=0.04), but the association attenuated to be insignificant after PSM adjusting for covariates (p=0.49). Other NIH Toolbox Cognition Battery measures (i.e. Flanker Inhibitory Control and Attention, List Sorting Working Memory, Pattern Comparison Process Speed) were not different between e-cigarette users and non-tobacco users.

**Table 2:** Propensity score modeling between exclusive past 6-month e-cigarette use at ages 12–13 years and NIH Toolbox Cognition Battery scores at follow-up after 1 year, ABCD Study, 2019–2022 (N=4574)^a^

*NIH Toolbox Cognition*	E-cigarette*		*Unadjusted^b^*			*PSM Adjusted ^c^*		
	*No use (N=4539)*	*Use (N=35)*	*b (SE)*	*95% CI*	*p*	*b (SE)*	*95% CI*	*p*
Flanker inhibitory control and attention	104.1 (0.3)	102.4 (1.5)	-1.7 (1.5)	-4.9–1.5	0.28	0.9 (1.3)	-1.9–3.7	0.51
List sorting working memory	105.7 (0.6)	101.5 (2.6)	-4.2 (2.6)	-9.7–1.3	0.12	-7.8 (4.5)	17.2–1.5	0.09
Oral reading recognition	99.4 (0.4)	97.0 (1.2)	-2.4 (1.2)	-4.8 – -0.003	0.05	-4.5 (1.3)	-7.3 – -1.7	0.003
Pattern comparison process speed	115.3 (0.7)	116.5 (3.7)	1.1 (3.4)	-6.1–8.3	0.74	-0.6 (6.8)	-14.9–13.6	0.93
Picture sequence memory	111.3 (0.6)	104.0 (3.5)	-7.3 (3.3)	-14.3 – -0.4	0.04	-6.3 (9.0)	-25.1–12.5	0.49
Picture vocabulary tests	93.8 (0.7)	89.9 (1.2)	-3.9 (1.0)	-5.9 – -1.8	0.001	-5.5 (0.7)	-7.1 – -3.9	<0.0001

* Data are given as weighted mean values with standard error.

a Sampling weights and site clustering were accounted for in all analyses. E-cigarette use is the predictive variable (independent) variable reported by youth at age of 12–13 years. Outcome (dependent) variables are NIH toolbox cognition measured 1 year later.

b Regression coefficients (b), standard errors (SE), 95% confidence intervals (CI) and p-value were estimated in the bivariate regression analyses using the original unmatched data set.

c Propensity score matching (PSM) was performed to balance 12 confounders, including age, sex at birth, race/ethnicity, youth pubertal stage, ever use of tobacco, past 6-month marijuana use, parental education level, parental income, parent monitoring, prenatal tobacco exposure, history of substance use, school environment factors, and child mental health assessed according to DSM-5 criteria. Regression coefficient b(SE) and p-value were estimated using the PSM matched data.

Table 3 presents traditional multivariable regression results for the Oral Reading Recognition and Picture Vocabulary Test scores. Puberty development scale and age were positively associated with both scores, and females had lower Picture Vocabulary Test scores than males (b [SE]= -1.1 [0.4]; 95% CI: -4.1 – -1.4; p=0.006). Compared to all White participants, Blacks had lower scores in both categories (b [SE]= -2.8 [0.7], 95% CI: -1.9 – -0.4; p<0.001; and b [SE]= -7.6 [0.6], 95% CI: -8.9 – -6.2; p<0.001, respectively), and Hispanics (b [SE]= -3.7 [0.3], 95% CI: -4.3 – -3.1; p<0.001) and other races (b [SE]= -1.2 [0.5], 95% CI: -2.3 – -0.2; p=0.02) had lower scores in Picture Vocabulary Test category. Parental education level and parental income were positively associated with Oral Reading Recognition and Picture Vocabulary Test scores, and the parent monitoring scale was also positively associated with the Picture Vocabulary Test score (b [SE]= 0.5 [0.2], 95% CI: 0.1–1.0; p=0.03). Self-reported past 6-month marijuana use was not significantly associated with the Reading Recognition and the Picture Sequence Memory scores at follow-up after 1 year.

**Table 3:** Multivariate regression analysis for cognitive functions between exclusive past 6-month e-cigarette use at ages 12–13 years and cognitive performance at follow-up after 1 year, ABCD Study, 2019–2022 (N=4574)^a^

*Variables*	*Oral reading recognition*			*Picture vocabulary test*		
	*b (SE) ^b^*	*95% CI*	*p*	*b (SE)*	*95% CI*	*p*
**Past 6-month e-cigarette use**						
No ®						
Yes	-2.7 (1.0)	-4.9 – -0.5	0.02	-3.0 (0.5)	-4.1 – -2.0	<0.001
**Puberty development scale**	0.5 (0.2)	0.1–0.9	0.01	0.8 (0.3)	0.2–1.3	0.009
**Age** (years)	0.7 (0.2)	0.3–1.1	0.002	1.1 (0.3)	0.5–1.6	0.001
**Sex**						
Male ®						
Female	-0.3 (0.3)	-1.0–0.3	0.32	-1.1 (0.4)	-1.9 – -0.4	0.006
**Race/ethnicity**						
White ®						
Black	-2.8 (0.7)	-4.1– -1.4	<0.001	-7.6 (0.6)	-8.9 – -6.2	<0.001
Hispanics	0.3 (0.7)	-1.1–1.7	0.68	-3.7 (0.3)	-4.3 – -3.1	<0.001
Other	0.5 (0.7)	-1.0–1.9	0.49	-1.2 (0.5)	-2.3 – -0.2	0.02
**Parental education level**						
High school or lower ®						
Some college or associate degree	1.3 (0.3)	0.7–1.9	<0.001	2.3 (0.3)	1.6–2.9	<0.001
College graduates	4.0 (0.4)	3.2–4.7	<0.0001	6.2 (0.4)	5.3–7.0	<0.001
**Parental income** ( $ )						
<50000 ®						
50000–99999	1.5 (0.4)	0.7–2.3	0.001	1.6 (0.4)	0.7–2.5	0.002
≥100000	1.7 (0.4)	0.9–2.5	<0.001	2.0 (0.5)	1.1–3.0	<0.001
**Parent monitoring scale**	0.4 (0.3)	-0.2–1.0	0.17	0.5 (0.2)	0.1–1.0	0.03
**Tobacco ever use**						
No ®						
Yes	-0.4 (1.7)	-3.9–3.1	0.81	-2.0 (1.4)	-4.9–1.0	0.18
**Past 6-month marijuana use**						
No ®						
Yes	0.8 (0.9)	-1.1–2.6	0.40	0.2 (2.1)	-4.2–4.6	0.92

a Multivariate regression analyses were performed with cognitive measures at follow-up after 1 year as the dependent variables. Sampling weights and site clustering were incorporated into the survey regression analytical procedures for statistical inference at the population level. The explanatory variables listed in column 1 were collected at baseline as simultaneous regressors.

b In the sensitivity analysis, we further adjusted additional covariates, including the number of e-cigarettes use days, prenatal tobacco exposure, history of substance use, school environment factors, and child mental health assessed according to DSM-5 criteria. E-cigarette use status was significantly associated with lower Oral Reading Recognition (adjusted b [SE]= -2.7 [1.0], p=0.0001) and Picture Vocabulary Test (adjusted b [SE]= -3.2 [0.9], p=0.0009). ® Reference categories.

## DISCUSSION

In this cohort study, the use of e-cigarettes in the past 6 months by young adolescents was associated with lower neurocognitive performance in two measures in the NIH Toolbox Cognition Battery Testing at follow-up after 1 year. To the best of our knowledge, this is one of the earliest studies to examine associations between neurocognitive performance and adolescent e-cigarette use.

Neurocognition plays a crucial role in adolescent development as it underpins essential processes such as learning, memory, problem-solving, and decision-making. These cognitive functions are vital not only for educational progress but also for the development of social skills, enabling adolescents to navigate complex social interactions and relationships effectively^23^. Adolescence is a critical period of development and social transition for children^24^. This prospective study identified two NIH Toolbox Cognition Battery categories associated with exclusive e-cigarette use: Oral Reading Recognition and Picture Vocabulary Tests. The Oral Reading Recognition^25^ task measures exposure to language materials and the cognitive skills involved in reading measures exposure to language materials as well as the cognitive skills involved in reading, while the Picture Vocabulary Test^26^ is designed to assess language and vocabulary comprehension, where study participants are asked to match the picture with the word. These assessments measure childhood language processing, a crucial skill for adolescents aged 12–14 years to gain successful educational and career advancement^27^.

Adolescent e-cigarette use continues to be a public health concern. Although recent national surveillance showed that the overall e-cigarette use prevalence among US adolescents peaked in 2019 and has declined since then, nicotine dependence related to e-cigarette use remains a concern, particularly among frequent users. Meanwhile, the current US e-cigarette market is dominated by high nicotine-strength e-cigarette products, with sales of high nicotine-level e-cigarettes increasing dramatically between January 2017 and March 2022^28^. Adolescent e-cigarette users exhibit significantly higher levels of exposure to nicotine metabolites (e.g. cotinine and trans-3'-hydroxycotinine) compared to non-tobacco users^29^. Urine levels of heavy metals such as lead and uranium have also been found to be higher among adolescent e-cigarette users compared to non-users of tobacco products^30^. Exposure to harmful and potentially harmful toxicants due to vaping during adolescence might contribute to lower neurocognitive performances in exclusive e-cigarette users reported in this study.

Our findings identified several risk and protective factors associated with adolescents’ language and reading skill development. Prior research suggests that even small differences in language- and reading-related cognitive skills during childhood and adolescence may be associated with later academic performance and educational trajectories, underscoring the need for cautious interpretation and further longitudinal investigation^23,31^. Consistent with previous literature^31^, puberty development stage and age are positively associated with neurocognitive performance; parental education, income, and parental monitoring serve as important protective factors, whereas racial minorities, particularly Black adolescents, scored significantly lower in the Oral Reading Recognition and Picture Vocabulary Test categories, after controlling for socioeconomic factors such as income and parental education level. These lower scores may indicate difficulties in reading fluency, word recognition, and overall language comprehension, which could impact academic performance and educational attainment during this critical developmental stage, addressing these challenges early on supports cognitive development and academic success in adolescence and beyond. The other NIH Toolbox tasks assessing attention, processing speed, and memory did not show significant differences, either due to small mean differences between groups or large variability. Since these tasks tap into distinct cognitive domains, the observed pattern appears to be domain-specific, particularly involving language-related skills, which may suggest selective vulnerability rather than generalized cognitive impairment. However, it can be noted that residual confounding may exist.

### Strengths and limitations

This study has several strengths, including a study design with sampling weight and clustering across 21 study sites over diverse geographical regions, the utilization of PSM to reduce biases from confounders, and objectively measured neurocognition assessments. In addition, we intentionally limited the exposure to exclusively use the e-cigarette in the past 6 months, thus avoiding confounding effects from other tobacco use.

This study has limitations. First, causal inference cannot be established based on this observational study. Although we applied the PSM that incorporated a number of covariates (e.g. sociodemographics, puberty, tobacco ever and past 6-month marijuana use) and excluded other tobacco use at baseline, there could be other factors that might impact cognitive measures and might be comorbid with e-cigarette use. For instance, parental IQ was not included as a covariate due to the lack of this variable in the ABCD study, even though genetic heritability research has shown its association with adolescent cognitive performance^32^. We mitigate this limitation by including parental education level, which is positively correlated with IQ in children^33^. Second, tobacco use status was self-reported, subject to social desirability biases, especially for younger respondents^34^. This could potentially lead to misclassification bias within the study. However, the test and retest reliability of self-reported behaviors related to tobacco use among adolescents is high^34^. Third, the sample size of exclusive e-cigarette users is relatively small, which might preclude statistical power in some analyses. We were also cautious not to over-control potential confounders, which might result in biased estimates and limit the effect size. Finally, only a subset of ABCD participants completed the Year 4 follow-up in the ABCD 5.0 data, resulting in a large reduction in the sample size from Year 3 substance use assessments to Year 4 neurocognitive assessment (~55%). This limited subset may limit the generalizability of this study, which could make applications to other populations more difficult. Further studies should assess the health effects of e-cigarette use when additional ABCD data are available.

## CONCLUSIONS

In this observational cohort study, exclusive e-cigarette use among early adolescents was associated with poorer performance in specific cognitive domains. These findings suggest that e-cigarette use may be linked to differences in neurocognitive performance and underscore the need for larger, longitudinal studies to clarify these associations further.

## Supplementary Material



## Data Availability

The data supporting this research are available from the ABCD study: https://nda.nih.gov/abcd
