# Supplementary material for: Propensity score modeling of adolescent e-cigarette use and cognitive performance: One-year follow-up study
Source: Tob Induc Dis. 2026 Mar 5;24:10.18332/tid/216705. doi: 10.18332/tid/216705 (PMC12964559; doi:10.18332/tid/216705)
Supplement: Supplementary file 1 [file TID-24-31-s1.pdf]

Appendix 1. NIH Toolbox Cognition Battery.

Outcome variables at 1-year follow-up was administered through an iPad, and tasks were completed within 35 minutes, including 1) *Picture Vocabulary Test* (PVT, Intraclass correlation [ICC]=0.81)[1] is designed to assess language and vocabulary comprehension where study participants are asked to match the picture with the word. 2) *Oral Reading Recognition (*ORR, ICC=0.97)[2]*:* the task measures exposure to language materials as well as the cognitive skills involved in reading measures exposure to language materials as well as the cognitive skills involved in reading. 3) *Pattern Comparison Processing Speed* (PCPS, ICC=0.84)[3] is to measure rapid visual process. Participants are shown two pictures and asked to determine by touch input whether the pictures are the same or not. The score is based on how many items they are able to complete correctly in a specific amount of time. 4) *Picture Sequence Memory* (PSM, ICC=0.76)[4] is designed to measure episodic memory. Participants need to reproduce the sequence of a series of fifteen pictures depicting activities or events that could occur in a particular setting. 5) *Flanker Inhibitory Control* (FIC, ICC=0.92)[5] is designed to assess executive function, attention, and inhibition. Participants need to make responses when surrounding stimuli and the target are either congruent or incongruent. 6) *List Sorting Working Memory* (LSWM, ICC=0.86)[6] is designed to measure working memory, categorization, and information processing. Children view pictures of animals or foods of varying sizes, with each picture accompanied by an auditory name from the iPad. They then repeat the items to the experimenter in order of size, from smallest to largest.

One NIH Toolbox task (Dimensional Charge Card Sort), along with the composite scores that were not assessed in the follow-up, are excluded from this study.

Appendix 2. Sensitivity Analysis

Due to the complexity of confounding in the association between e-cigarette use and cognitive development, other covariates in sensitivity analysis further incorporated prenatal tobacco exposure, youth self-reported ever substance use (e.g., marijuana, alcohol, and other illicit drugs, such as cocaine,

methamphetamine, ecstasy/MDMA, ketamine, gamma-hydroxybutyrate, heroin, psilocybin, salvia, other hallucinogens; anabolic steroids, inhalants, prescription stimulants, sedatives, opioid pain relievers, and OTC cough/cold medicine). Youth who reported using any of these substances were classified as positive, while those with no reported use were classified as negative. Childhood school environment was assessed by School Risk & Protective Factors (SRPF) subscale consisting of 6 items (range: 6-24) on perceptions of safety, teacher-student relationships, clear rules, respectful peer interactions, inclusiveness, and opportunities for extracurricular involvement (Cronbach's alpha=.62).[7] Child mental health was assessed by the Diagnostical and Statistical Manual of Mental Disorders, 5th edition(DSM-5) with the sum of six DSM-5-oriented diagnostic categories (affective problems, anxiety problems, somatic problems, attention-deficit/hyperactivity disorder(ADHD), oppositional defiant problems, conduct problems, Cronbach's alpha=.83). This child behavioral checklist (CBCL) subscale utilizes a 3-point Likert scale where 0 indicates the symptom is absent, 1 indicates it occurs sometimes, and 2 indicates it occurs often.

In the sensitivity analysis, we further adjusted additional covariates, including the number of e-cigarette use days, history of substance use, school environment factors, and child mental health assessed according to DSM-5 criteria. E-cigarette use status was significantly associated with lower Oral Reading Recognition (adjusted Regression coefficients (b) [Standard Error (SE)]= -3.8 [-0.1]; 95% CI = [-5.6, -2.2]; p=0.0001) and Picture Vocabulary Test (adjusted b [SE]= -3.5 [-0.04]; 95% CI = [-5.4, -1.6]; p=0.0009). Other NIH Toolbox Cognition batteries were not significant.

Appendix 3. Propensity Score Modeling

Due to the ethical restrictions, researchers are unable to randomize study participants into e-cigarette use versus no use. Instead, the observational studies are leveraged with a realistic design to capture various characteristics among study participants. Systematic sample differences between e-cigarette users and non-users may bias estimates of cognitive outcomes. Factors such as socioeconomic status, parental education, mental health, and other substance use can influence both the likelihood of using e-cigarettes and cognitive performance, making it difficult to isolate the effect of e-cigarette use alone. Propensity score modeling (PSM) offers a principled framework to draw matched analytical samples for those with or without e-cigarette use by modeling the probability of e-cigarette use as a function of observed covariates and using this probability to construct comparable groups.[8] The utilization of PSM can balance the covariate distribution between e-cigarette users and non-users, thus reducing sample bias and decreasing the likelihood of confounding factors from observational data.[9]

We performed the propensity score modeling (PSM) to reduce potential confounding in estimating the average treatment effect (ATE) of exclusive e-cigarette use on cognitive outcomes.

Firstly, using the PROC PSMATCH procedure in SAS, we conducted a logistic regression, where the treatment group was defined as youth who reported exclusive e-cigarette use and non-tobacco users served as the control group. The explanatory variables included12 confounders: age, sex at birth, race/ethnicity, youth pubertal stage, ever use of tobacco, past 6-month marijuana use, parental education, parental income, parent monitoring, prenatal tobacco exposure, history of substance use, school environment factors, and child mental health assessed according to DSM-5 criteria. These covariates were selected based on a review of the literature, established cognitive assessment protocols, and factors known to influence child cognitive development, brain growth, and patterns of tobacco use.[10-13]

Secondly, matching was conducted using a variable ratio method (k=1 to 4) with a caliper of 0.5 on the logit of the propensity score. Balance diagnostics were assessed using standardized differences, cumulative distribution function (CDF) plots, and box plots for covariates. The final matched sample was weighted using ATE weights generated by PROC PSMATCH, and these were multiplied by the original ABCD sampling weights to produce the final analysis weight.

Thirdly, weighted linear regression models were then performed using PROC SURVEYREG, adjusting for the clustered sampling design (site-level clustering) to evaluate the associations between exclusive e-cigarette use and six cognitive outcomes: attention, working memory, recognition memory, processing speed, sequence memory, and vocabulary. Each model specified the cognitive domain as the outcome and exclusive e-cigarette use as the primary independent variable.

Appendix 4. Improvement in the balance between exclusive e-cigarette users and non-tobacco users before and after propensity score modeling (PSM) analysis.

Variable	Observations	Mean Difference	Standard Deviation	Percent Reduction
**Logit Propensity Score**	**All**	2.6	1.6	
	**Matched**	0.8		70.4
	**Weighted Matched**	0.0		99.9
**Puberty**	**All**	0.1	0.7	
	**Matched**	-0.1		18.2
	**Weighted Matched**	-0.2		0.0
**Age, year**	**All**	0.4	0.6	
	**Matched**	0.0		86.8
	**Weighted Matched**	0.0		97.8
**Parental monitoring**	**All**	-0.4	0.5	
	**Matched**	-0.1		73.1
	**Weighted Matched**	-0.1		85.0
**School environment**	**All**	-2.0	3.3	
	**Matched**	-0.2		90.9
	**Weighted Matched**	0.7		66.2
**DSM-5**	**All**	9.2	14.0	
	**Matched**	2.6		71.4
	**Weighted Matched**	0.2		98.3
**Sex**	**All**	0.0	0.5	
	**Matched**	0.0		42.6
	**Weighted Matched**	0.0		0.0
**Tobacco ever use**	**All**	0.0	0.1	
	**Matched**	0.0		23.6
	**Weighted Matched**	0.0		100.0
**Marijuana use (past 6 month)**	**All**	-0.2	0.3	
	**Matched**	-0.1		31.3
	**Weighted Matched**	0.0		79.8
**Substance ever use**	**All**	-0.3	0.5	
	**Matched**	-0.1		77.2
	**Weighted Matched**	0.0		85.7
**Prenatal tobacco exposure**	**All**	0.0	0.3	
	**Matched**	0.0		0.0
	**Weighted Matched**	0.1		0.0

All: differences before the PSM; Matched: differences in the matched sample; Weighted Matched: differences applying the weight in the matched sample.

References

1. Gershon RC, Cook KF, Mungas D, et al. Language measures of the NIH Toolbox Cognition Battery. *J Int Neuropsychol Soc*. Jul 2014;20(6):642–51. doi:10.1017/S1355617714000411
2. Gershon RC, Slotkin J, Manly JJ, et al. IV. NIH Toolbox Cognition Battery (CB): measuring language (vocabulary comprehension and reading decoding). *Monogr Soc Res Child Dev*. Aug 2013;78(4):49–69. doi:10.1111/mono.12034
3. Carlozzi NE, Beaumont JL, Tulsky DS, Gershon RC. The NIH Toolbox Pattern Comparison Processing Speed Test: Normative Data. *Arch Clin Neuropsychol*. Aug 2015;30(5):359–68. doi:10.1093/arclin/acv031
4. Mantey DS, Case KR, Omega-Njemnobi O, Springer AE, Kelder SH. Use frequency and symptoms of nicotine dependence among adolescent E-cigarette users: Comparison of JUUL and Non-JUUL users. *Drug Alcohol Depend*. Nov 1 2021;228:109078. doi:10.1016/j.drugalcdep.2021.109078
5. Zelazo PD, Anderson JE, Richler J, Wallner-Allen K, Beaumont JL, Weintraub S. II. NIH Toolbox Cognition Battery (CB): measuring executive function and attention. *Monogr Soc Res Child Dev*. Aug 2013;78(4):16–33. doi:10.1111/mono.12032
6. Tulsky DS, Carlozzi NE, Chevalier N, Espy KA, Beaumont JL, Mungas D. V. NIH toolbox cognition battery (CB): Measuring working memory. *Monographs of the Society for Research in Child Development*. 2013;78(4):70–87.
7. Arthur MW, Briney JS, Hawkins JD, Abbott RD, Brooke-Weiss BL, Catalano RF. Measuring risk and protection in communities using the Communities That Care Youth Survey. *Eval Program Plann*. May 2007;30(2):197–211. doi:10.1016/j.evalprogplan.2007.01.009
8. Lanehart RE, de Gil PR, Kim ES, Bellara AP, Kromrey JD, Lee RS. Propensity score analysis and assessment of propensity score approaches using SAS procedures. 2012:
9. Haukoos JS, Lewis RJ. The Propensity Score. *JAMA*. Oct 20 2015;314(15):1637–8. doi:10.1001/jama.2015.13480
10. Dai HD, Doucet GE, Wang Y, et al. Longitudinal Assessments of Neurocognitive Performance and Brain Structure Associated With Initiation of Tobacco Use in Children, 2016 to 2021. *JAMA Netw Open*. Aug 1 2022;5(8):e2225991. doi:10.1001/jamanetworkopen.2022.25991
11. Dai HD, Pierce J, Beseler C, et al. Hierarchical Modeling of Psychosocial, Parental, and Environmental Factors for Susceptibility to Tobacco Product Use in 9-10-Year-Old Children. *J Adolesc Health*. Feb 2023;72(2):267–276. doi:10.1016/j.jadohealth.2022.09.021
12. Puga TB, Dai HD, Wang Y, Theye E. Maternal Tobacco Use During Pregnancy and Child Neurocognitive Development. *JAMA Network Open*. 2024;7(2):e2355952–e2355952.
13. Hackman DA, Cserbik D, Chen JC, et al. Association of Local Variation in Neighborhood Disadvantage in Metropolitan Areas With Youth Neurocognition and Brain Structure. *JAMA pediatrics*. Aug 1 2021;175(8):e210426. doi:10.1001/jamapediatrics.2021.0426

© 2026 Dai H.D. et al.
